# Is social capital higher in areas with a higher density of historic assets? Analyses of 11,112 adults living in England

**DOI:** 10.1177/17579139221145609

**Published:** 2023-02-12

**Authors:** HW Mak, E Gallou, D Fancourt

**Affiliations:** Department of Behavioural Science and Health, Institute of Epidemiology & Health Care, University College London, London, UK; Historic England, Policy and Evidence, London, UK; Department of Behavioural Science and Health, Institute of Epidemiology & Health Care, University College London, London WC1E 7HB, UK

**Keywords:** social capital, heritage engagement, Historic England, Index of Multiple Deprivation

## Abstract

**Aims:**

Previous evidence suggests that engagement with heritage such as visiting heritage sites provides benefits for people’s mental and social wellbeing, and helps to establish social capital. However, far less is known about whether living in areas of historic built environment also helps build social capital. Furthermore, it remains unclear how the association between historic built environment and social capital may vary across heritage engagement frequency and areas of deprivation levels. This study was therefore designed to explore the cross-sectional relationship between historic built environment and social capital.

**Methods:**

Analysis was based on three datasets: Understanding Society: The UK Household Longitudinal Study Waves 5 (2013/2015) and 6 (2014/2016), 2019 National Heritage List for England, and 2015 English Index of Multiple Deprivation (IMD). Ordinary least squares (OLS) regressions were applied to estimate the relationships between historic built environment (listed buildings, scheduled monuments, and registered parks and gardens) and social capital (personal relationships, social network support, civic engagement, and trust and cooperative norms).

**Results:**

We found that people living in places with greater historic built environment experienced higher levels of personal relationships, social network support, and civic engagement. However, these associations were attenuated once rurality was adjusted. Individuals living in areas of greater levels of historic built environment displayed higher levels of trust and cooperative norms, even after adjusting for all relevant covariates. Heritage engagement frequency was found to moderate the association between historic built environment and personal relationships. Similarly, IMD was also found to moderate the association between historic built environment and trust and cooperative norms.

**Conclusion:**

These findings highlight the importance of neighbourhood environment in building social capital in communities. Particularly, areas with heritage assets may provide both socially inviting and aesthetically pleasing environments that could help strengthen community and restore pride in place.

## Introduction

Heritage sites, including historic listed buildings, scheduled monuments such as Roman remains, castles, bridges, burial mounds, and the remains of deserted villages and industrial sites, and registered historic parks and gardens, are recognised as a valuable asset for individual and community wellbeing.^1^ Evidence has suggested that heritage helps reduce levels of anxiety and mood disorder, provides a sense of pride, and creates opportunities for social integration and community engagement.^1^ In England, there are over 400,000 list entries, 95% of which are listed buildings, 5% are scheduled monuments, and 0.4% are historic parks and gardens (those with a special historic interest or designed historic landscapes with outstanding importance and rarity).^1,2^ These heritage sites receive substantial levels of engagement from the public, with around 74.5 million visits made to historic properties in England each year,^3^ and over 7 in 10 adults and 2 in 3 children and young people visiting a heritage site at least once every 12 months.^4,5^

Engagement in heritage has been shown to provide benefits for people’s mental and social wellbeing. For instance, research has found that engaging in heritage once a year to three to four times a year or more are associated with improvements in life satisfaction, greater mental health functioning, and reduced mental distress.^6–10^ Moreover, heritage-based cultural activities in museums have been found to relate to positive emotions, a sense of empowerment and confidence, improved social connectiveness and interactions, and reduced social isolation.^11–13^ Some potential key ingredients for the associations include new spaces for conversation and friendship to develop, a source of distraction from negative emotions, and inspiration and pride in artwork and craftwork.^13^ Furthermore, visiting historic landscapes and parks have been shown to help enhance self-esteem and alleviate feelings of anger and depression.^14^ Specific studies of interventions designed to connect people with heritage have shown similar results. For example, the Human Henge intervention, a programme designed to engage people living in England who experienced mental health problems with historic landscapes, was found to help improve their mental health wellbeing and connections with others (*N* = 35).^15^ Similarly, an intervention involving handling and discussion of heritage objects in hospital and healthcare settings (including acute and elderly care, residential, and psychiatric) in England found increased levels of positive affect, wellness and happiness, and reduced negative affect among older adults aged 65–85 with chronic conditions, anxiety and depression (*N* = 40).^16^ Mental and social wellbeing benefits of heritage involvement may also arise from volunteering in settings such as historic houses and gardens.^17^

In addition to these wellbeing benefits, other research has highlighted the effects of heritage engagement on social capital. Social capital is a multidimensional construct that can broadly be distinguished into three dimensions: structural, relational, and cognitive social capital.^18,19^ Structural social capital refers to network ties and membership of or participation in party organisations, religious association, or other voluntary organisations. Relational social capital, on the other hand, refers to personal relationships and interactions which often involve shared history, respect, trust, obligations, identity, and emotional attachments. Finally, the cognitive dimension refers to shared norms, values, and interpretations.^19^ These three dimensions are highly interconnected and are suggested to help facilitate cooperation and connections within community.^18,19^ Various benefits of social capital have been identified. For instance, it has been found that, in Canada, trust in people and institutions such as the police, health care system, banks, and a strong social network of friends were associated with happiness.^20^ A study on a Chinese population has found similar findings:^21^ trust was positively correlated with self-reported general health, psychological health, and life satisfaction, while holding socio-economic and demographic characteristics in constant. This was possibly explained by emotional support, shared information on social and health services, and control of deviant behaviours. Notably, the effects of trust on life satisfaction were similar in size to the effects of household income (both with a beta coefficient of 0.3).^21^ Moreover, it has been suggested that, while civic engagement membership in party-affiliated organisations was related to better self-reported general health, membership in voluntary organisations helped support better psychological health and improve life satisfaction.^21^ On a community level, social capital is essential for neighbourhood development as it helps facilitate coordination and cooperation for community benefits and encourages civic actions within a community.^22,23^ Conversely, a deficit of social capital can have compound negative effects of social and health inequalities as well as social unrest.^24,25^

In the past two decades, social capital has been one of the policy priorities in the UK across different disciplines, including public health, urban planning, economy, and community development. The role of it has particularly been highlighted in the recent ‘levelling up’ White Paper published in 2022,^26^ which emphasises on improving living standards and quality of life, especially in more deprived areas, promoting equality and opportunity, strengthening community and local leadership, and restoring pride in place; all core components of social capital.^26^ In seeking to build social capital, the place where people live becomes important as it can help create a social environment which enables and facilitates residents’ interaction with other community members.^27,28^ In particular, places with historic elements usually operate as cultural attractions, providing additional incentives for people to engage in face-to-face socialising, to connect with people from different cultural and socio-economic clusters, and to cultivate a stronger sense of place provided by its cultural distinctiveness and uniqueness.^29^

Previous research has identified three broad social and community benefits of heritage that help form social capital: (i) greater interactions between people through activities such as participating in heritage-type activities or engaging in other kinds of unrelated interactions such as dog walking, (ii) a deeper sense of collective identity and sense of place (e.g. sharing knowledge about the past), and (iii) enhanced levels of awareness and understanding of other community members and hence facilitate community cohesion.^30,31^ However, most of the research to date has focused on heritage engagement (i.e. visiting heritage sites for days out). Far less is known about whether living in areas of historic built environment, where historic assets serve as a setting for daily community life, helps build social capital, even if one does not specifically set aside time to engage with specific heritage sites.

Indeed, existing literature has hinted that areas with higher concentration of heritage assets may produce a stronger sense of place, in addition to creating social environment that enables people to meet and interact, as they help provide the historic elements that define a neighbourhood character.^32,33^ For instance, qualitative research findings from the UK and Poland with participants coming from a range of age and ethnic groups have shown that places with heritage characteristics provide a source of identity and local pride and can facilitate communal activities such as local field walking groups.^33,34^ For some heritage assets such as monuments, historic markets, and heritage parks and gardens, they can act as a landmark for people to meet socially,^31^ as well as facilitating social mixing in diverse communities.^2,33^ Furthermore, heritage buildings with their intricate architectural styles and designed infrastructure provide visual aesthetic and sensory experiences that could lead to improvements in wellbeing,^35^ as well as encouraging people to engage in outdoor recreation.^33^ Leaving home offers opportunities for social encounters that could help maintain loose ties between neighbours and promote community integration.^33^ These positive effects were also found in urban areas that were characterised by green areas and a predominance of historic properties, where residents reported greater neighbourhood satisfaction of walkability, feelings of safety, and less pollution and stress.^36^ Finally, historic built environment may offer opportunities for residents to meet and contribute to decisions and active place-making, shaping collectively the character of the place they live with other members of their community (e.g. improvements in quality of shopping streets, regeneration and open spaces). This could encourage people getting involved in local activities such as fund raising, elections of planning representatives, and local archaeology projects.^32^ A survey of over 2400 adults from YouGov (2017) has also revealed that people living in conservation areas are more likely to engage in development and planning decisions in their local areas than those living outside of them,^37^ although it should be noted that the survey might contain self-selection bias.

However, historic built environment and its social impacts are likely to be geographically patterned, particularly in places where there are interventions and funding to develop local economic and tourism-related activities as well as regeneration programmes to help maintain those assets. Geographical factors are likely related to the socio-economic characteristics of individuals living in the catchment areas, the influence of those factors on people’s cultural behaviours and social wellbeing such as deprivation and safety levels, and area-specific social processes such as social contagion and networks.^38,39^ Yet it remains unclear whether the association between historic built environment and social capital varies across neighbourhoods, as has been reflected in other parallel studies, which found that the effects of heritage and culture engagement on mental health and life satisfaction might be more prominent in areas of higher deprivation.^6^ Understanding how the impact of historic built environment may vary geographically has implications on urban planning and social policies, which aim to enhance social capital within communities facing barriers to development due to deprivation through existing place-based resources such as heritage and cultural buildings and infrastructures.

Therefore, this article explored three interconnected research questions (RQs):

*RQ1.* Is historic built environment associated with social capital (defined by four sub-scales: personal relationships, social network support, civic engagement, and trust and cooperative norms)?
*RQ2.* Is the association between historic built environment and social capital independent of, or moderated by, the amount that individuals engage with heritage (measured by frequency of visits)?
*RQ3.* Does the association between historic built environment and social capital vary by neighbourhood deprivation?

To address these RQs, we used three different datasets: (1) Understanding Society: The UK Household Longitudinal Study (UKHLS) Waves 5 (2013/2015) and 6 (2014/2016), (2) 2019 National Heritage List for England (the official, up to date, registration of all nationally protected historic buildings and sites in England [1]), and (3) 2015 English Index of Multiple Deprivation (IMD), and applied statistical regressions to estimate the cross-sectional relationships between historic built environment and social capital while accounting for potential confounding factors. This study focused on historic built environment in both urban and rural areas.

## Data and Methods

Data from the UKHLS follow over 50,000 individuals from 30,000 households annually and collect rich information about respondents’ socio-demographics; community group engagement; social, mental, and physical wellbeing; as well as their relationships within neighbourhoods.^40^ In this study, we extracted a sample of adults living in England who responded to both Waves 5 (2013/2015; response rate = 85%), where cultural and heritage engagements were measured, and 6 (2014/2016; response rate = 84%), where social capital was measured (*N* = 36,809). We only considered respondents providing data across all measures (*N* =25,185).

To investigate the role of historic built environment, we used geo-coded UKHLS data in which participating households’ addresses were matched to neighbourhood zones. Neighbourhoods were defined as 2011 census lower super output areas (LSOAs). LSOAs are designed for the consistent reporting of small area statistics in England and Wales. Using the 2011 LSOA geocodes, we attached the 2019 National Heritage List for England data on heritage assets, which provides geodata for all nationally protected historic buildings and sites in England. We used data from 2019 as the data were more maturely developed. Providing that these assets are historical, the historic built environment does not differ substantially within a decade. The data include battlefields, listed buildings (2.5% are listed as Grade I, 5.8% as Grade II*, and 91.7% as Grade II), parks and gardens with historic characteristics, protected wreck sites, scheduled monuments, World Heritage Sites, and conservation areas. The database is official and regularly updated which indicates the exact location of the protected sites, buildings, and areas, as well as basic textual information across various fields, including the type of Grade for buildings and parks. More information can be found in Historic England^41^ database.

In the present study, we considered three types of historic assets that are commonly found in local neighbourhoods: listed buildings (Grades I, II*, and II; *N* =371,843), scheduled monuments (*N* = 26,207), and registered parks and gardens (*N* = 3490; the number exceeds the official number due to multiple entries as such assets often extend to more than one local authority districts (LAD) given the size of them).^2^

**Diagram 1.**
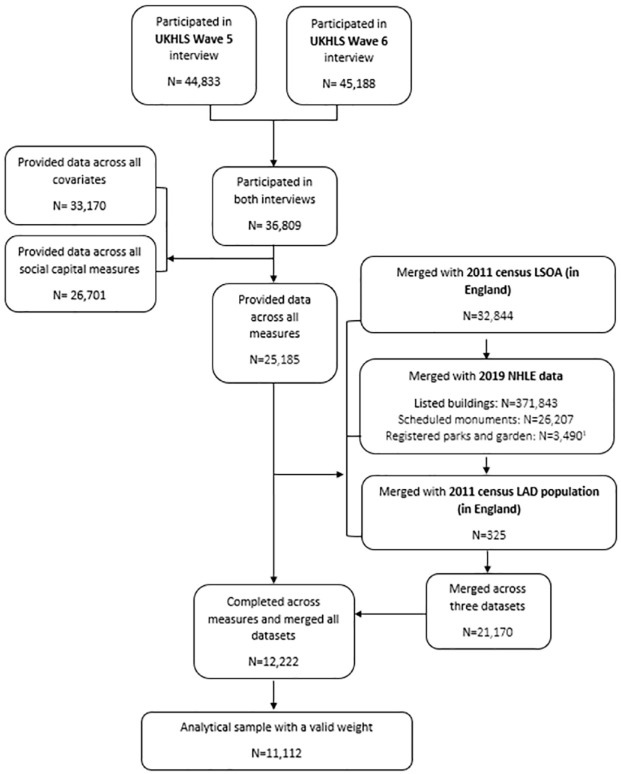
A flowchart of analytical sample. ^1^The number exceeds the official number due to multiple entries as such assets often extend to more than one local authority districts (LAD) given the size of them. UKHLS: Understanding Society: The UK Household Longitudinal Study; NHLE: National Heritage List for England; LSOA: lower super output areas.

In addition, to explore the role of neighbourhood deprivation, we further attached the 2015 IMD data, which use a range of input datasets to rank the relative deprivation of LSOAs across seven weighted domains: income, employment, health deprivation and disability, education, skill and training, crime, barriers to housing and services, and living environment. After matching, the number of survey participants was 12,222. A flowchart of analytical sample is indicated in Diagram 1.

### Measures

#### Historic built environment

Historic built environment measured in 2019 was defined as the number of heritage assets (listed buildings, scheduled monuments, and registered parks and gardens) across LAD per thousand people and was categorised into five groups according to the data from the Heritage Indicators 2020 published by Historic England:^2^ 0–1.4, 1.5–3.4, 3.5–7.3, 7.4–14.8, and 14.9+. Balance across these categories was achieved, making it suitable for subsequent analyses. The measure was used as a continuous measure given the normally distributed pattern across the five categories (Supplemental Figure S1).

#### Social capital

We considered four key aspects of social capital measured in 2014/2016: personal relationships, social network support, civic engagement, and trust and cooperative norms based on the UKHLS measures proposed by the Office for National Statistics.^42,43^ For *personal relationships*, two measures were considered: number of close friends (a 6-point scale, ranging from 0 to 5+) and proportion of respondents who agree on regularly stopped and talked with people within neighbourhood (a 5-point scale, ranging from strongly disagree to strongly agree). These two measures were positively although weakly correlated (*r* = .12). They were combined to a single indicator and were computed additively with a range of 1–10.

For *social network support*, two indicators were considered: whether or not respondents provided special help to at least one sick, disabled, or elderly person living or not living with them (yes versus no), and whether people felt they could borrow things from neighbours (a 5-point scale, ranging from strongly disagree to strongly agree). To enable the measures to be more consistent, we collapsed the latter measure to binary: agree/strongly agree versus neither/disagree/strongly disagree. These two measures were weakly correlated (tetrachoric correlation = 0.03) and were summed with a range of 0–2.

Regarding *civic engagement*, respondents were asked if they were members of organisations, whether political, voluntary, professional, or recreational (yes versus no), and whether they regularly volunteered (a 10-point scale, ranging from never to 3 or more days a week collapsed to binary: at least once a month versus less than once a month or never). These two measures were positively correlated (tetrachoric correlation = .53) and were then summed with a range of 0–2.

Finally, for *trust and cooperative norms*, three measures were considered: the extent to which people in the neighbourhood (1) could be trusted, (2) were willing to help their neighbours, and (3) felt sense of belonging in the neighbourhood. These measures were on a 5-point scale and were positively correlated (ranging from *r* = .43 to *r* = .60). They were computed additively with a range of 3–15.

Given that all four social capital variables were measured on different scales, they were subsequently standardised to have a mean of 0 and a standard deviation of 1.

A set of covariates that were shown important and relevant to social capital in previous empirical studies were considered in the model. They were all measured in baseline collected in UKHLS Wave 5 (2013/2015) and included age, gender (female versus male), ethnicity (white ethnic versus ethnic minorities), cohabitating status (living with a partner versus not living with a partner, including those who were single, divorced/separated, or widowed), employment status (employed versus not employed including the unemployed, retired, students, etc.), education (with degree versus without degree), total personal monthly gross income (including from labour, miscellaneous, private benefit, investment, pension, and social benefit; quartiles), presence of parent(s) in the household (yes versus no), presence of child(ren) in the household (yes versus no), and frequency of cultural attendance such as visiting a play/drama or a ballet performance (a continuous variable with a 5-point scale, ranging from none in the past year to at least once a month). We have additionally considered rurality defined in 2011 census LSOA identification codes in our models (living in rural areas versus not living in rural areas).

### Analysis

RQ1: To understand the relationship between historic built environment and social capital, we ran a cross-sectional analysis using ordinary least squares (OLS) regression models. Given that historic built environment can be highly correlated with personal demographic and socio-economic factors (e.g. a preference for living in places with historical characteristics), the regression models were constructed sequentially to understand the changes of the association between historic built environment and each of the social capital measures. Model 1 was unadjusted. In Model 2, we additionally adjusted for demographic backgrounds: age, gender, ethnicity, cohabitating status, presence of parent(s) in the household, and presence of child(ren) in the household. In Model 3, we additionally controlled for socio-economic positions (SEP): education, employment status, income, and cultural attendance frequency. Finally, in Model 4, we further adjusted for rurality. As a sensitivity analysis, all models were repeated by restricting the respondents to those who did not move houses between Waves 5 and 6 interviews (*N* = 10,490) to account for potential changes in historic built environment exposure during the follow-up period.

RQ2: To explore whether the relationship between historic built environment and social capital was independent of heritage engagement frequency (a continuous variable with a 5-point scale, ranging from none to at least once a month), we repeated the analysis while additionally adjusting for levels of heritage engagement. To understand whether the relationship was moderated by the levels of engagement given that areas with more historical assets may attract more visits from the local people, we further included an interaction term (historic built environment × heritage engagement levels) in the analysis. Number of observations of each interaction cell is provided in Supplemental Table S1.

RQ3: Finally, to understand whether the relationship between historic built environment and social capital varied by levels of area deprivation (a decile scale, 1 being most deprived 10% and 10 being least deprived 10%), we tested the interaction effect (historic built environment × IMD) in our full model. Number of observations of each interaction cell is provided in Supplemental Table S2.

All models were weighted using inverse probability weights derived from Waves 5 and 6 weights supplied with UKHLS. These weights have been tailored to the analytical sample and should correct our estimates by taking into account differential sample selection and retention probabilities. Missingness was handled using list-wise deletion. This gives a core sample size of 11,112 (participants with a valid weight).

## Results

In our sample, the average age was 48 years. 52% were female, 93% were of White ethnic background, and around 64% of the participants were living with a partner. In addition, 14% of the respondents were living with at least one parent in the household and 29% living with their children. Around 3 in 10 respondents did not visit heritage sites in the past year (in line with the figure of heritage visits presented in the Department of Digital, Culture, Media & Sport 2019/2020 report [5]), whereas 1 in 10 visited at least once a month (Supplemental Table S3(a)). In general, the average age was slightly higher in areas with highly concentrated historic buildings (Supplemental Table S3(b)).

### RQ1: is historic built environment associated with social capital?

Our results show that, after adjusting for demographic backgrounds and SEP, living in places with greater historic built environment was associated with higher levels of personal relationships (coef = 0.03, 95% confidence interval (CI) = 0.01, 0.04; beta = 0.04), social network support (coef = 0.02, 95% CI = 0.01, 0.04; beta = 0.03), and civic engagement (coef = 0.02, 95% CI = 0.01, 0.04; beta = 0.03) (Table 1; Model 3). However, these associations were attenuated after adjusting for rurality (Table 1; Model 4). Nonetheless, the association was maintained across all models for historic built environment and greater trust and cooperative norms (coef = 0.03, 95% CI = 0.01, 0.04; beta = 0.04) (Table 1; Model 4). Results were replicated when restricting the sample to those who did not move houses between the two interview waves (Supplemental Table S4).

**Table 1:** OLS regression estimating the association between historic built environment and social capital (*N* = 11,112)

	Personal relationships			Social network support			Civic engagement			Trust and cooperative norms		
	Coef.	95% CI	*p*-value	Coef.	95% CI	*p*-value	Coef.	95% CI	*p*-value	Coef.	95% CI	*p*-value
Model 1: adjusted for historic built environment only	**0.05**	**0.03, 0.06**	**0.000**	**0.04**	**0.02, 0.05**	**0.000**	**0.04**	**0.02, 0.05**	**0.000**	**0.08**	**0.07, 0.10**	**0.000**
Model 2: Model 1 + demographic backgrounds	**0.03**	**0.01, 0.05**	**0.000**	**0.02**	**0.01, 0.04**	**0.006**	**0.02**	**0.01, 0.04**	**0.004**	**0.06**	**0.05, 0.08**	**0.000**
Model 3: Model 2 + socio-economic position	**0.03**	**0.01, 0.04**	**0.000**	**0.02**	**0.01, 0.04**	**0.005**	**0.02**	**0.01, 0.04**	**0.001**	**0.06**	**0.05, 0.08**	**0.000**
Model 4 (full model): Model 3 + rurality	0.00	−0.01, 0.02	0.617	0.00	−0.01, 0.02	0.663	0.02	−0.00, 0.03	0.059	**0.03**	**0.01, 0.04**	**0.003**

OLS: ordinary least squares; CI: confidence interval; SEP: socio-economic position.

Demographic factors included age, gender, ethnicity, cohabiting status, whether or not living with children, and whether or not living with parents. SEP factors included education level, employment status, total personal monthly gross income, and cultural engagement frequency. Rurality indicates whether respondents were living in rural areas. Bold values denote statistical significance at the *p* < 0.05 level.

### RQ2: is the association independent of, or moderated by, heritage engagement frequency?

Given that rurality appeared to have absorbed a large amount of variation in the relationship between historic built environment and social capital measures, we performed two sets of models: before and after adjusting for rurality. Before adjusting for rurality, historic built environment was positively associated with personal relationships (coef = 0.03, 95% CI = 0.01, 0.04; beta = 0.04), social network support (coef = 0.02, 95% CI = 0.00, 0.04; beta = 0.03), civic engagement (coef = 0.02, 95% CI = 0.00, 0.03; beta = 0.03), and trust and cooperative norms (coef = 0.06, 95% CI = 0.05, 0.08; beta = 0.09) even when accounting for the frequency of heritage engagement (Table 2). However, after considering rurality, the associations with personal relationships, social network support, and civic engagement were attenuated (Table 3). For trust and cooperative norm, the pattern was different. Historic built environment continued to relate to higher levels of the outcome even independent of heritage engagement levels and rurality (coef = 0.02, 95% CI = 0.01, 0.04; beta = 0.03) (Table 3).

**Table 2:** OLS regression estimating the association between historic built environment and social capital with an inclusion of heritage engagement level as both a covariate and moderator: not adjusting for rurality (*N* = 11,112).

	Personal relationships			Social network support			Civic engagement			Trust and cooperative norms		
	Coef.	95% CI	*p*-value	Coef.	95% CI	*p*-value	Coef.	95% CI	*p*-value	Coef.	95% CI	*p*-value
Historic built environment	**0.03**	**0.01, 0.04**	**0.001**	**0.02**	**0.00, 0.04**	**0.011**	**0.02**	**0.00, 0.03**	**0.009**	**0.06**	**0.05, 0.08**	**0.000**
Interaction terms												
Historic built environment	**0.05**	**0.02, 0.08**	**0.001**	0.02	−0.01, 0.04	0.156	0.01	−0.01, 0.03	0.329	**0.07**	**0.04, 0.10**	**0.000**
Engagement level	**0.10**	**0.06, 0.14**	**0.000**	0.03	−0.01, 0.07	0.114	**0.08**	**0.04, 0.11**	**0.000**	**0.07**	**0.03, 0.11**	**0.001**
Historic built environment × engagement level	−**0.01**	−**0.02**, −**0.00**	**0.032**	0.00	−0.01, 0.01	0.853	0.00	−0.01, 0.01	0.396	−0.01	−0.02, 0.01	0.288

OLS: ordinary least squares; CI: confidence interval.

The models were adjusted for demographic factors, socio-economic position, and heritage engagement level. Bold values denote statistical significance at the *p* < 0.05 level.

**Table 3:** OLS regression estimating the association between historic built environment and social capital with an inclusion of heritage engagement level as both a covariate and moderator: adjusting for rurality (*N* = 11,112).

	Personal relationships			Social network support			Civic engagement			Trust and cooperative norms		
	Coef.	95% CI	*p*-value	Coef.	95% CI	*p*-value	Coef.	95% CI	*p*-value	Coef.	95% CI	*p*-value
Historic built environment	0.00	−0.02, 0.02	0.898	0.00	−0.02, 0.02	0.822	0.01	−0.01, 0.03	0.199	**0.02**	**0.01, 0.04**	**0.008**
Interaction terms												
Historic built environment	0.02	−0.01, 0.05	0.106	0.00	−0.03, 0.03	0.974	0.00	−0.02, 0.03	0.814	**0.04**	**0.01, 0.07**	**0.021**
Engagement level	**0.10**	**0.06, 0.14**	**0.000**	0.03	−0.01, 0.07	0.107	**0.08**	**0.04, 0.11**	**0.000**	**0.07**	**0.03, 0.11**	**0.001**
Historic built environment × engagement level	**−0.01**	**−0.02, −0.00**	**0.029**	0.00	−0.01, 0.01	0.878	0.00	−0.01, 0.01	0.405	−0.01	−0.02, 0.00	0.260

OLS: ordinary least squares; CI: confidence interval.

The models were adjusted for demographic factors, socio-economic position, heritage engagement level, and rurality. Bold values denote statistical significance at the *p* < 0.05 level.

**Figure 1:**
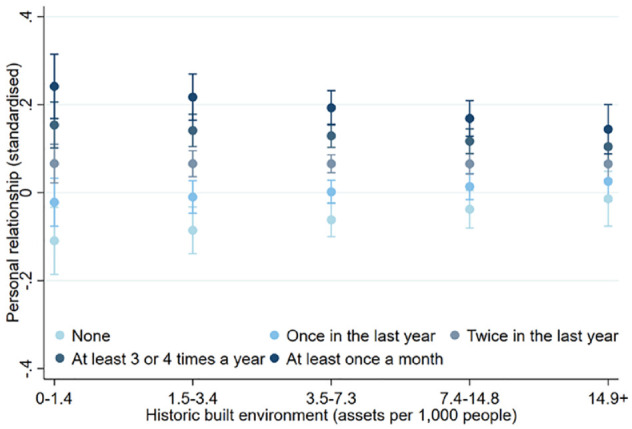
Historic built environment and personal relationships by heritage engagement level

When examining the interacting effects of historic built environment × engagement frequency, a moderating effect was found for personal relationships regardless of whether rurality was adjusted (coef = −0.01, 95% CI = −0.02, −0.00; beta = −0.07) (Tables 2 and 3). This suggests that the association between heritage engagement levels and personal relationships may be less salient for people living in areas with greater historic built environment, and that the differences in personal relationships between people with higher and lower heritage engagement rates may also be reduced in areas with greater historic built environment (Figure 1). In contrast, no moderating associations were found for social network support, civic engagement, or trust and cooperative norms.

### RQ3: does the association vary by neighbourhood deprivation?

**Figure 2:**
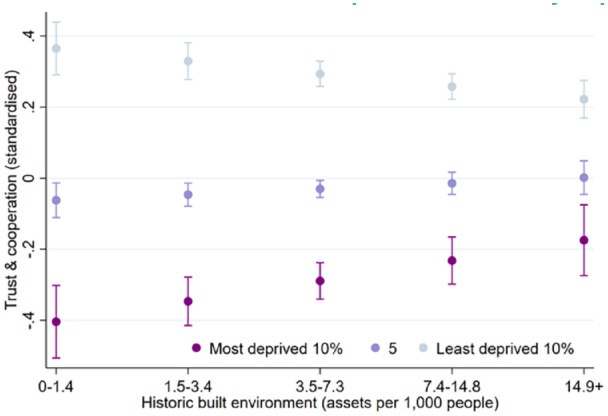
Historic built environment and trust and cooperative norms by deprivation level

After adjusting for all covariates, we found a moderating effect of IMD on the association between historic built environment and trust and cooperative norms (coef = −0.01, 95% CI = −0.02, −0.00; beta = −0.14) (Table 4). The result suggests that differences in trust and cooperative norms between higher and lower levels of area deprivation may be reduced in areas with greater historic built environment (Figure 2). No moderating associations were found for personal relationships, social network support, and civic engagement.

**Table 4:** OLS regression estimating the moderating effect of levels of area deprivation on the association between historic built environment and social capital (*N* = 11,112).

	Personal relationships			Social network support			Civic engagement			Trust and cooperative norms		
	Coef.	95% CI	*p*-value	Coef.	95% CI	*p*-value	Coef.	95% CI	*p*-value	Coef.	95% CI	*p*-value
Interacting with IMD												
Historic built environment	0.02	−0.02, 0.07	0.293	0.01	−0.03, 0.06	0.529	−0.00	−0.04, 0.04	0.920	**0.07**	**0.02, 0.12**	**0.008**
IMD	**0.06**	**0.04, 0.08**	**0.000**	0.01	−0.01, 0.03	0.278	0.01	−0.00, 0.03	0.129	**0.10**	**0.07, 0.12**	**0.000**
Historic built environment × IMD	−0.01	−0.01, 0.00	0.093	−0.00	−0.01, 0.00	0.540	0.00	−0.00, 0.01	0.666	**−0.01**	**−0.02, −0.00**	**0.003**

OLS: ordinary least squares; CI: confidence interval; IMD: Index of Multiple Deprivation.

The models were adjusted for demographic factors, socio-economic position, and rurality. Bold values denote statistical significance at the *p* < 0.05 level.

## Discussion

This was the first study combining three different datasets, namely UKHLS Waves 5 (2013/2015) and 6 (2014/2016), National Heritage List for England (2019), and English IMD (2015), to examine the association between historic built environment and social capital. Our results show that people living in places with greater historic built environment experienced higher levels of personal relationships, social network support, and civic engagement, even after considering levels of heritage engagement, but these associations were attenuated once rurality was factored in. However, individuals living in areas of greater levels of historic built environment displayed higher levels of trust and cooperative norms. This relationship was persistent irrespective of demographic factors, SEP, rurality, and the amount of people engaging in heritage activities. Furthermore, differences in trust and cooperative norms between higher and lower levels of area deprivation were lower in areas with greater historic built environment, yet caution should be taken given that areas that are densely packed with historic assets also have lower levels of area deprivation.

The results are in parallel to previous studies which explored the association between historic built environment and social capital.^32,34^ In particular, we found that the effects of historic built environment on personal relationships, social network support, and civic engagement might be partly explained by rurality of the areas. Previous studies have provided evidence that people are happier, more supportive, and trusting if there is green space in the neighbourhoods,^44,45^ suggesting that physical attributes of places play a role in people’s social wellbeing. This could be explained by the correlation between exposure to green space and healthy behaviours such as walking, community gardening, and cycling.^46,47^ These behaviours may provide opportunities for residents to interact with their neighbours and to engage in social activities with them, which in turn facilitates relationships within local communities, and increases senses of neighbourhood safety and trusting. There was also some evidence that when comparing a predominately built-up area with historic and green elements and an urban park, their effects on participants’ affective and restorative outcomes were similar,^48^ supporting their development in social capital. Yet areas with historic sites and characteristics may additionally facilitate community belonging and identity. Indeed, environmental psychologists have acknowledged the significance of both physical environment and people’s perceptions of and experiences in the environment, such as place attachment,^49^ and heritage assets may help enhance positive perceptions and experiences.^1^

However, our findings also indicated that rurality may not explain fully the association between historic built environment and all types of social capital. We found that historic built environment was significantly associated with greater levels of trust and cooperative norms even after considering rurality and heritage engagement. One explanation for this could be that, in addition to social environment, historic built environment also provides an aesthetically pleasing environment for people living in the catchment area to engage more in outdoor activities with a perception of safety and hence increase social interactions within the area.^50,51^ Additionally, the historically and culturally meaningful experiences for residents living in historic neighbourhoods may contribute to intercultural understanding and development of trust between groups from diverse backgrounds.^31,52^ On a related note, places with heritage characteristics may also provide a sense of uniqueness and place attachment through shared historic roots.^1,31,53^ In addition, historic built environment could provide a vehicle for the local community to collaborate and work together on projects about the historic assets in the area.^1^ For instance, a case study in Bellingham Heritage Centre showed that a group of local volunteers ran multiple projects in preserving local buildings and artefacts. This has attracted schools close by to visit the Heritage Centre every year for young people to understand more about their local area and heritage, and has attracted funding to support the Centre’s continued development^1^ and create a cultural pole for the area. This suggests that people living in areas of greater historic built environment may be more likely to be exposed to community activity opportunities that help strengthen their sense of belonging and form social capital.

Our study additionally explored whether the association between historic built environment and social capital varied by levels of heritage engagement and area of deprivation. We found that, for instance, the differences in personal relationships between people with higher and lower heritage engagement rates may be reduced in areas with greater historic built environment. Particularly, among those who did not engage in heritage, their personal relationships increased as historic built environment indexes increased. In contrast, people who engaged at least once a month may have poorer personal relationships if they lived in areas with highly concentrated historic buildings (versus areas of less concentrated historic buildings). Similar patterns were found for the relationship between historic built environment, area of deprivation, and trust and cooperative norms, where historic built environment may have a positive effect on the social outcome in more deprived areas but a negative effect in less deprived areas. Several factors may explain this. Historic built environment places are likely to attract tourism (especially in affluent places with well-maintained historic buildings and infrastructures) that may interrupt the formation of social capital for local residents.^34^ For instance, for people who engage in heritage regularly, it is possible that mass tourism may have led to the loss of symbolic meaning of heritage assets and thus taken away the sense of pride and uniqueness of the areas from the local community residents.^34^ In addition, the costs and benefits from heritage-related tourism may not necessarily be equally distributed. It is plausible that while some community members may enjoy the benefits tourism brings such as income, others may suffer from the costs of it such as restricted use of historical public space for local recreational use.^34^ The unequal distribution of benefits may worsen the relationship among community members.^34^ However, for people who never or rarely engaged in heritage or for people living in more deprived areas, living in places of historic built environment may enable them to increase satisfaction of their neighbourhood, to feel safe, and thus to participate more in outdoor activities where they could interact with other community members.^36^ They may also benefit from the aesthetic experiences which provide a therapeutic effect for residents to support and maintain their wellbeing,^35^ making it easier for them to initiate communications with neighbours. Such findings have potential policy implications relating to the ‘levelling-up’ agenda, in which the government could make use of the existing heritage sites and infrastructures (e.g. launching upskilling projects such as heritage conservation or education projects, or investing in long-term heritage-led regeneration projects) to support the development of trust and cooperation among people living in more deprived areas who often have fewer cultural opportunities,^54^ while looking to build the sustainability of areas already experiencing high levels of tourism.

This study has a number of strengths, including the use of nationally representative survey data merging with nationally listed historic assets data provided by Historic England and 2015 English IMD, which enabled an investigation about the role of historic built environment while controlling for important variables such as heritage engagement (measured by number of visits made to historic assets) and rurality of the living area. However, the study is not without limitations. While our analysis involved estimating the levels of historic built environment of participants’ living areas, we were not able to control for participants’ residential preferences on their interests in history. It is possible that participants who were interested in heritage might choose to reside in areas of greater historic built environment and hence were likely to form social connections with others who shared the same interests. Furthermore, although we have avoided bias relating to people’s understanding and perception of ‘historic environment’ by objectively estimating the density of historic assets across local authority districts, we were unable to take into account the quality, value, and function of those assets.^31^ For instance, preferences to use such assets may be affected by levels of maintenance and physical condition of those assets. Future work is required to further explore the quality, in addition to quantity, of heritage sites and infrastructures.

While our study considered heritage sites commonly found in local neighbourhoods, sites that were not nationally protected (e.g. traditional houses or buildings within conservation areas that are not listed) were not included in the analysis due to data complexity which might be prone to measurement errors.^55^ Moreover, our measures for social capital may reflect more on bonding social capital (i.e. social connections between individuals who share similar values, norms, demographic backgrounds, attitudes, personal characteristics, etc.) than bridging social capital (i.e. connections between individuals who are dissimilar in relation to background and characteristics). Future research is required to explore whether historic built environment also helps facilitate bridging social capital. Finally, we only considered participants whose household LSOAs were matched to the LSOAs of heritage sites. This means that individuals who lived in proximity to those sites but not in exact LSOAs might have a different level of historic built environment compared to those who matched successfully. More sophisticated geographical data analysis that takes into account of the distance between residential areas and heritage assets is needed. Further research is also required to better examine whether people living in the catchment area were likely to engage in heritage locally or whether they might travel to different neighbourhoods where more heritage resources and opportunities were more readily available.

## Conclusion

There is a growing consensus that social capital helps communities to thrive and to be more resilient, and that heritage may help build social capital. Overall, our study shows that living in areas of greater historic built environment helps improve personal relationships, social network support, civic engagement, and trust and cooperative norms, with some of the associations potentially being explained by rurality of the area (which has also been shown to provide opportunities for the development of social capital). Furthermore, the associations between historic built environment and personal relationships and trust and cooperative norms may be moderated by the rate of heritage engagement and neighbourhood deprivation, with people with lower engagement rate and those living in more deprived areas may benefit more from living in areas with higher historic built environment levels. These findings highlight the importance of neighbourhood environment in building social capital in communities. Particularly, areas with heritage assets may provide both socially inviting and aesthetically pleasing environments that could encourage outdoor and social activities, providing opportunities for interactions with neighbours, facilitating learning and discussions around shared heritage locations, as well as supporting joint civic action in projects around heritage in the area. These activities could help enhance a sense of belonging and trust in neighbourhood. For individuals living in areas with low levels of historic built environment, it is crucial to ensure that they have equal access to historic assets (e.g. through local trips, reduced entrance fees in paid sites, or promoting family and group visits for infrequent visitors). The local communities and councils are also encouraged to share knowledge on the historic background and character of the area to strengthen neighbourhood connections and trust through a shared sense of history and roots (e.g. London Underground has been displaying its heritage in recent years to yield public interest in the tube history), with additional avenues to individual and community wellbeing improvements.

## Supplemental Material

sj-pdf-1-rsh-10.1177_17579139221145609 – Supplemental material for Is social capital higher in areas with a higher density of historic assets? Analyses of 11,112 adults living in England Supplemental material, sj-pdf-1-rsh-10.1177_17579139221145609 for Is social capital higher in areas with a higher density of historic assets? Analyses of 11,112 adults living in England by HW Mak, E Gallou and D Fancourt in Perspectives in Public Health
