# Supplementary material for: Is social capital higher in areas with a higher density of historic assets? Analyses of 11,112 adults living in England
Source: Perspect Public Health. 2023 Feb 12;144(4):251–62. doi: 10.1177/17579139221145609 (PMC11308256; doi:10.1177/17579139221145609)
Supplement: sj-pdf-1-rsh-10.1177_17579139221145609 – Supplemental material for Is social capital higher in areas with a higher density of historic assets? Analyses of 11,112 adults living in England [file sj-pdf-1-rsh-10.1177_17579139221145609.pdf]

Supplementary materials

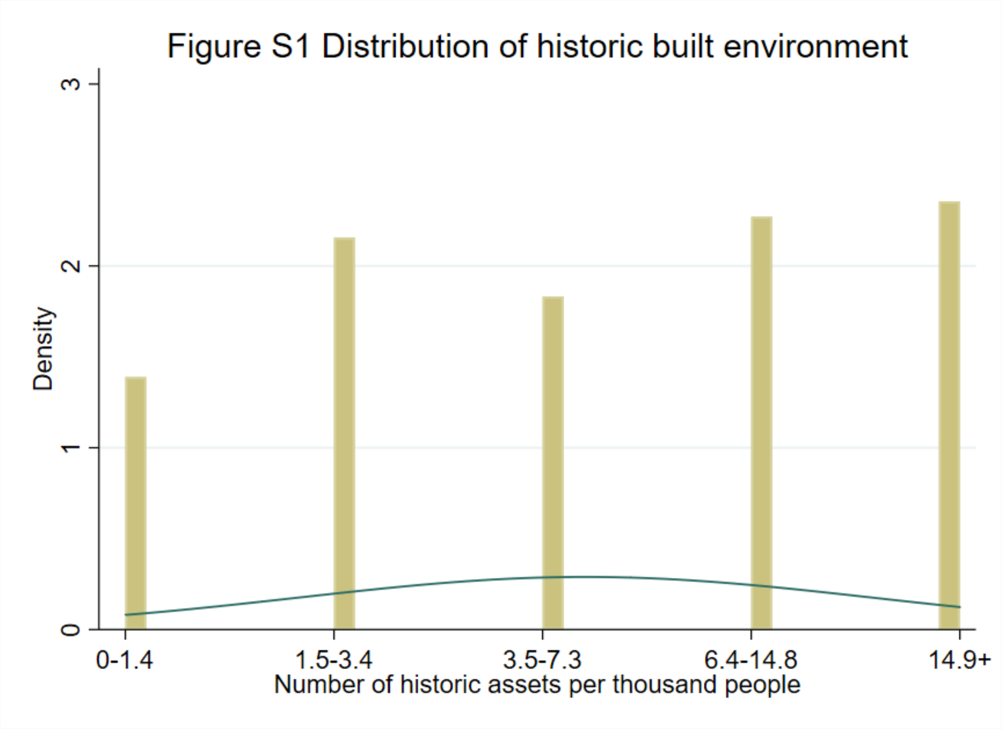

Table S1 Number of observations in each cell in the interaction terms between historic built environment and frequency of heritage engagement (N=11,112)

Historic built environment	Frequency of heritage engagement	Frequency	Percentage
0-1.4	None in the past year	630	5.15
0-1.4	Once in the past year	205	1.68
0-1.4	Twice in the past year	251	2.05
0-1.4	At least 3 or 4 times a year	435	3.56
0-1.4	At least once a month	176	1.44
1.5-3.4	None in the past year	814	6.66
1.5-3.4	Once in the past year	356	2.91
1.5-3.4	Twice in the past year	383	3.13
1.5-3.4	At least 3 or 4 times a year	781	6.39
1.5-3.4	At least once a month	300	2.45
3.5-7.3	None in the past year	681	5.57
3.5-7.3	Once in the past year	298	2.44
3.5-7.3	Twice in the past year	340	2.78
3.5-7.3	At least 3 or 4 times a year	648	5.30
3.5-7.3	At least once a month	271	2.22
7.4-14.8	None in the past year	706	5.78
7.4-14.8	Once in the past year	338	2.77
7.4-14.8	Twice in the past year	421	3.44
7.4-14.8	At least 3 or 4 times a year	951	7.78
7.4-14.8	At least once a month	359	2.94
14.9+	None in the past year	684	5.60
14.9+	Once in the past year	281	2.30
14.9+	Twice in the past year	402	3.29
14.9+	At least 3 or 4 times a year	1008	8.25
14.9+	At least once a month	503	4.12

Table S2 Number of observations in each cell in the interaction terms between historic built environment and Index of Multiple Deprivation (IMD) (N=11,112)

Historic built environment	IMD	Frequency	Percentage
0-1.4	1-Most deprived	156	1.28
0-1.4	2	248	2.03
0-1.4	3	200	1.64
0-1.4	4	202	1.65
0-1.4	5	166	1.36
0-1.4	6	168	1.37
0-1.4	7	156	1.28
0-1.4	8	163	1.33
0-1.4	9	155	1.27
0-1.4	10-Least deprived	83	0.68
1.5-3.4	1-Most deprived	355	2.90
1.5-3.4	2	278	2.27
1.5-3.4	3	291	2.38
1.5-3.4	4	262	2.14
1.5-3.4	5	210	1.72
1.5-3.4	6	258	2.11
1.5-3.4	7	287	2.35
1.5-3.4	8	249	2.04
1.5-3.4	9	216	1.77
1.5-3.4	10-Least deprived	228	1.87
3.5-7.3	1-Most deprived	183	1.50
3.5-7.3	2	182	1.49
3.5-7.3	3	189	1.55
3.5-7.3	4	189	1.55
3.5-7.3	5	194	1.59
3.5-7.3	6	257	2.10
3.5-7.3	7	281	2.30
3.5-7.3	8	265	2.17
3.5-7.3	9	261	2.14
3.5-7.3	10-Least deprived	237	1.94
7.4-14.8	1-Most deprived	80	0.65
7.4-14.8	2	101	0.83
7.4-14.8	3	136	1.11
7.4-14.8	4	230	1.88
7.4-14.8	5	256	2.09
7.4-14.8	6	281	2.30
7.4-14.8	7	425	3.48
7.4-14.8	8	348	2.85
7.4-14.8	9	423	3.46
7.4-14.8	10-Least deprived	495	4.05
14.9+	1-Most deprived	16	0.13
14.9+	2	36	0.29
14.9+	3	120	0.98
14.9+	4	259	2.12
14.9+	5	422	3.45
14.9+	6	538	4.40
14.9+	7	407	3.33
14.9+	8	391	3.20
14.9+	9	369	3.02

14.9+	10-Least deprived	320	2.62

Table S3a Descriptive statistics of the analytical sample (N=11,112) and the excluded sample

	Weighted analytical sample	Raw excluded sample
	Mean (SE) / %	Mean (SE) / %
Age		*N=24,587*
	48.1 (0.19)	48.8 (0.12)
Gender		*N=24,584*
Female	51.8%	53.5%
Male	48.2%	46.5%
Ethnicity		*N=23,654*
White ethnic	93.1%	85.0%
Ethnic minorities	6.94%	15.0%
Cohabiting status		*N=24,587*
Living with a partner	63.6%	46.8%
Not living with a partner	36.4%	53.2%
Employment status		*N=22,168*
Employed	60.4%	53.5%
Not employed	39.6%	46.5%
Education		*N=24,451*
With degree	28.0%	21.9%
Without degree	72.0%	78.2%
Total personal monthly gross income		*N=24,587*
£0-£687	23.6%	24.2%
£687-£1304	22.3%	26.8%
£1304-£2193	24.6%	25.5%
£2193-£26433	29.5%	23.6%
Presence of parent(s) in the household		*N=24,587*
Yes	14.3%	15.1%
No	85.7%	84.9%
Presence of child(ren) in the household		*N=24,587*
Yes	29.4%	24.9%
No	70.6%	75.1%
Frequency of cultural attendance		*N=21,448*
None in the past year	19.9%	29.3%
Once in the past year	10.4%	9.78%
Twice in the past year	15.2%	13.9%
At least 3 or 4 times a year	36.4%	30.5%
At least once a month	18.1%	16.6%
Rurality		*N=5,417*
Living in rural areas	30.0%	24.6%
Not living in rural areas	70.0%	75.4%
Historic built environment (number of assets per thousand people)		*N=5,417*
0-1.4	14.4%	14.3%
1.5-3.4	21.1%	24.9%
3.5-7.3	18.0%	20.1%
7.4-14.8	22.8%	19.9%
14.9+	23.7%	20.8%
Frequency of heritage engagement		*N=21,440*
None in the past year	28.6%	41.4%
Once in the past year	12.7%	12.6%
Twice in the past year	14.9%	13.2%

At least 3 or 4 times a year	30.8%	23.8%
At least once a month	13.1%	8.99%
Levels of areas deprivation (a decile scale, 1 being most deprived 10% and 10 being least deprived 10%)		*N=5,417*
	6.11 (0.03)	5.44 (0.04)
Social capital		
Personal relationships (ranging from 1-10)		*N=20,162*
	7.54 (0.02)	7.38 (0.01)
Social network support (ranging from 0-2)		*N=16,375*
	0.70 (0.01)	0.66 (0.01)
Civic engagement (ranging from 0-2)		*N=21,506*
	0.74 (0.01)	0.64 (0.00)
Trust and cooperative norms (ranging from 3-15)		*N=19,825*
	11.5 (0.02)	11.4 (0.01)

Table S3b Descriptive statistics by levels of historic built environment (weighted; N=11,112)

Historic built environment (number of assets per thousand people)	0-1.4 (N=1,505)	1.5-3.4 (N=2,360)	3.5-7.3 (N=2,030)	7.4-14.8 (N=2,540)	14.9+ (N=2,677)
	Mean (SE) / %	Mean (SE) / %	Mean (SE) / %	Mean (SE) / %	Mean (SE) / %
Age	45.7 (0.54)	46.2 (0.41)	48.0 (0.45)	48.5 (0.39)	51.0 (0.38)
Gender					
Female	51.6%	52.4%	52.3%	51.6%	51.2%
Male	48.4%	47.6%	47.8%	48.4%	48.8%
Ethnicity					
White ethnic	81.7%	90.7%	92.9%	97.2%	98.2%
Ethnic minorities	18.3%	9.32%	7.08%	2.83%	1.80%
Cohabiting status					
Living with a partner	61.6%	58.9%	63.7%	63.4%	69.2%
Not living with a partner	38.4%	41.1%	36.3%	36.6%	30.8%
Employment status					
Employed	62.3%	60.9%	60.6%	60.0%	59.1%
Not employed	37.7%	39.1%	39.4%	40.0%	40.9%
Education					
With degree	29.8%	30.0%	27.4%	26.8%	26.7%
Without degree	70.2%	70.0%	72.6%	73.2%	73.3%
Total personal monthly gross income					
£0-£687	22.4%	24.6%	25.4%	23.0%	22.8%
£687-£1304	20.6%	21.9%	21.6%	21.4%	25.0%
£1304-£2193	25.8%	24.2%	25.2%	24.2%	24.3%
£2193-£26433	31.3%	29.4%	27.9%	31.5%	27.9%
Presence of parent(s) in the household					
Yes	17.6%	16.1%	14.6%	13.6%	11.0%
No	82.4%	83.9%	85.4%	86.4%	89.0%
Presence of child(ren) in the household					
Yes	33.1%	29.1%	28.0%	29.3%	28.7%
No	66.9%	70.9%	72.0%	70.7%	71.3%
Frequency of cultural attendance					
None in the past year	19.7%	20.6%	22.8%	18.1%	18.8%

Once in the past year	9.02%	10.9%	10.0%	11.1%	10.4%
Twice in the past year	16.3%	14.3%	13.3%	17.3%	14.8%
At least 3 or 4 times a year	36.3%	35.3%	35.5%	35.8%	38.9%
At least once a month	18.6%	19.0%	18.4%	17.7%	17.1%
Rurality					
Living in rural areas	0.82%	7.34%	26.6%	36.7%	64.1%
Not living in rural areas	99.2%	92.7%	73.4%	63.3%	35.9%
Frequency of heritage engagement					
None in the past year	34.9%	30.1%	29.9%	25.2%	25.8%
Once in the past year	11.9%	14.4%	13.4%	13.1%	10.6%
Twice in the past year	14.5%	15.1%	15.6%	15.2%	14.0%
At least 3 or 4 times a year	27.0%	29.2%	28.9%	33.7%	33.2%
At least once a month	11.7%	11.2%	12.2%	12.9%	16.4%
Levels of areas deprivation (a decile scale, 1 being most deprived 10% and 10 being least deprived 10%)	5.24 (0.08)	5.33 (0.07)	6.06 (0.07)	6.86 (0.05)	6.67 (0.04)
Social capital					
Personal relationship (ranging from 1-10)	7.29 (0.06)	7.46 (0.05)	7.49 (0.04)	7.68 (0.04)	7.65 (0.04)
Social network support (ranging from 0-2)	0.67 (0.02)	0.67 (0.01)	0.67 (0.02)	0.73 (0.01)	0.75 (0.01)
Civic engagement (ranging from 0-2)	0.71 (0.02)	0.71 (0.02)	0.70 (0.02)	0.77 (0.01)	0.80 (0.01)
Trust and cooperative norms (ranging from 3-15)	11.1 (0.07)	11.3 (0.05)	11.5 (0.05)	11.6 (0.04)	11.8 (0.04)

Table S4 OLS regression estimating the relationship between historic built environment and social capital amongst individuals who did not move houses between the two interviews (N=10,490)

	Personal relationships			Social network support			Civic engagement			Trust and cooperative norms		
	Coef.	95%CI	P -value	Coef.	95%CI	P -value	Coef.	95%CI	P -value	Coef.	95%CI	P -value
Model 1: adjusted for historic built environment only	**0.05**	**0.03, 0.06**	**0.000**	**0.04**	**0.02, 0.05**	**0.000**	**0.04**	**0.02, 0.06**	**0.000**	**0.09**	**0.07, 0.10**	**0.000**
Model 2: Model 1 + demographic backgrounds	**0.03**	**0.01, 0.04**	**0.001**	**0.02**	**0.01, 0.04**	**0.006**	**0.02**	**0.01, 0.04**	**0.004**	**0.07**	**0.05, 0.08**	**0.000**
Model 3: Model 2 + socio-economic position	**0.03**	**0.01, 0.04**	**0.001**	**0.02**	**0.01, 0.04**	**0.005**	**0.02**	**0.01, 0.04**	**0.001**	**0.07**	**0.05, 0.08**	**0.000**
Model 4 (full model): Model 3 + rurality	0.00	-0.02, 0.02	0.876	0.00	-0.01, 0.02	0.597	0.02	-0.00, 0.03	0.065	**0.03**	**0.01, 0.05**	**0.001**

Note: Demographic factors included age, gender, ethnicity, cohabiting status, whether or not living with children, and whether or not living with parents. SEP factors included education levels, employment status, total personal monthly gross income and cultural engagement frequency. Rurality indicates whether respondents were living in rural areas.
